# Titanium Nanobowl-Based Nest-Like Nanofiber Structure Prepared at Room Temperature and Pressure Promotes Osseointegration of Beagle Implants

**DOI:** 10.3389/fbioe.2022.841591

**Published:** 2022-02-24

**Authors:** Lei Sun, Xuzhuo Chen, Haizhang Mu, Yin Xu, Ruiguo Chen, Rong Xia, Lunguo Xia, Shanyong Zhang

**Affiliations:** ^1^ Department of Oral and Maxillofacial Surgery, School and Hospital of Stomatology, Cheeloo College of Medicine, Shandong University and Shandong Key Laboratory of Oral Tissue Regeneration and Shandong Engineering Laboratory for Dental Materials and Oral Tissue Regeneration, Jinan, China; ^2^ Department of Stomatology, The Second Affiliated Hospital of Anhui Medical University, Hefei, China; ^3^ Shanghai Key Laboratory of Stomatology, Department of Oral Surgery, College of Stomatology, Ninth People’s Hospital, Shanghai Research Institute of Stomatology, National Clinical Research Center of Stomatology, Shanghai Jiao Tong University School of Medicine, Shanghai, China; ^4^ Laboratory of Molecular Neuropsychology, School of Mental Health and Psychological Sciences, Anhui Medical University, Hefei, China; ^5^ High Magnetic Field Laboratory, CAS Key Laboratory of High Magnetic Field and Ion Beam Physical Biology, Hefei Institutes of Physical Science, Chinese Academy of Sciences, Hefei, China; ^6^ Department of Orthodontics, Collage of Stomatology, Ninth People’s Hospital, Shanghai Jiao Tong University School of Medicine, Shanghai, China

**Keywords:** titanium, nanofiber, biocompatibility, osseointegration, implant

## Abstract

Nest-like nanofiber structures have potential applications in surface modifications of titanium implants. In this study, nest-like nanofiber structures were prepared on a titanium surface at room temperature and pressure by using the nanobowl template-assisted method combined with alkali etching. The characterization and biocompatibility of this material were analyzed by cellular adhesion, death, CCK-8, ALP, and RT-PCR assays *in vitro,* and osseointegration was evaluated by micro-CT and fluorescent labeling *in vivo*. The results showed that this nest-like nanofiber structure has a firmer and asperate surface than nanotubes, which leads to better cellular adhesion, proliferation, and differentiation capacity. In a beagle alveolar bone implant model, the nest-like nanofiber structure showed a better osseointegration capacity. In conclusion, this nest-like nanofiber structure has potential applications in dental implantology.

## 1 Introduction

Dental implantation, one of the most effective methods of repairing oral dentition defects and dentition loss, restores the morphology and function of patients’ oral and maxillofacial systems (Elani et al., 2018; Guglielmotti et al., 2019). Medical pure titanium has become the preferred material for artificial implants because of its metal properties, corrosion resistance, biocompatibility, and bone-bonding properties (Marin et al., 2013; Shubin Wang et al., 2020; Matter et al., 2021). There is usually no direct contact between the titanium implant and the bone, although a layer of fiber tissue is formed, which leads to poor osseointegration and implant failure (Xia et al., 2018). Therefore, surface modification of the titanium metal surface is necessary (Qingge Wang et al., 2020; Lu et al., 2020; Xue et al., 2020).

There are various methods for surface modification of titanium, such as grit blasting (Qingge Wang et al., 2020), acid etching (Han et al., 2019; Nobre et al., 2020), electrochemical anodic oxidation (Cho et al., 2015), hydrothermal method (Vishnu et al., 2019), sol-gel (Xue et al., 2020), and plasma spraying (Ding et al., 2016), which can lead to different micro- and nanostructures on the surface of the titanium. Two or more methods can also be combined to make special structures on the surface of the titanium. Maher et al. combined metal selective laser melting (SLM), electrochemical anodization, and hydrothermal (HT) methods to create vertically arranged sharp bionic nanostructures on the surface of Ti6Al4V. These structures can increase the deposition of hydroxyapatite minerals in simulated body fluids (SBF) and the adhesion of human osteoblast-like cells (NHBCs) (Maher et al., 2021). Recently, various tube-, ball-, slide-, rod-, and fiber-like microstructures have been developed in order to increase the osseointegration of implants (Chi et al., 2007; Ou and Lo, 2007). Wang et al. studied TiO_2_ nanotubes with different diameters (30, 70, and 100 nm). The results showed that compared with pure titanium implants, the expression of *OSX*, *Col-I*, and *ALP* increased in three groups of TiO_2_ nanotubes increased, and the fluorescence labeling of bones around the implants was more significant, with 70-nm-diameter nanotubes showing best effects (Wang et al., 2011). Jiang et al. prepared titanium nano-agglomerates and titanium nanorods and three kinds of micro- and nanostructures that all show enhanced protein adsorption capacity, viability, adhesion, and differentiation capacity of bone marrow mesenchymal stem cells. Among them, nanograss fiber structures have the best biological compatibility (Chi et al., 2007). Lin et al. have shown that the nano-/micro-nest-like structure and the nanotube structure have better osseointegration capacity than the nanosponge structure (Lin et al., 2014). All these results indicate that the nest-like structure has good biocompatibility and promotes osseointegration.

Currently, the nest-like structure with a uniform and controllable structure on the surface of the titanium implant is mainly prepared by using the hydrothermal method in KOH solution (Anitha et al., 2015; Lee et al., 2015; Wandiyanto et al., 2020)or NaOH solution (Chi et al., 2007; Lin et al., 2014), most of which are hydrothermally treated at 110–150°C for 2–24 h, and some need to be calcined at 450–500°C for 2–4 h (Chi et al., 2007; Lin et al., 2014; Anitha et al., 2015; Lee et al., 2015; Wandiyanto et al., 2020). The hydrothermal method uses an aqueous solution as the reaction system in a specific sealed reactor such as an autoclave. A high-temperature and high-pressure environment can be created by heating and pressurizing the water solution, which dissolves and recrystallizes the insoluble titanium (Kang et al., 2020). Although the hydrothermal method has advantages of controlling the size and morphology of titanium oxide particles and microporous materials, it has a long reaction cycle, restricts equipment requirements (high temperature- and high pressure-resistant steel, corrosion-resistant liner), has technical difficulties such as strict temperature and pressure control, and is relatively costly (Chen et al., 2017; Cui et al., 2017). Of note, the nest-like nanostructure prepared by the hydrothermal method is bottom-up, which is attached to the surface of the titanium substrate (Anitha et al., 2015) and creates a weak interactive binding force that limits the stability. Therefore, it is urgent to develop a simple, easier-to-operate, safe, and controllable method to prepare nest-like nanofiber structures with a higher binding force to the titanium substrate under room temperature and normal pressure. With the development of nanotechnology in recent years, it has been shown that nanoscale morphology can significantly expand a specific surface area (Kim et al., 2012; Shin et al., 2016; Li et al., 2017; Ourari et al., 2019) to increase the chemical reaction rate (Hou et al., 2018; Hamans et al., 2020; Zhang et al., 2020). Shin et al. reported that 70-nm titanium nanoparticles can be etched in a KOH solution to prepare a titanium nest-like structure at room temperature, which is based on the mechanism of those nanoparticles that have a higher specific surface area (Shin et al., 2016) and increase the effective contact area of KOH and TiO_2_ and reduce the reaction temperature.

**FIGURE 1:**
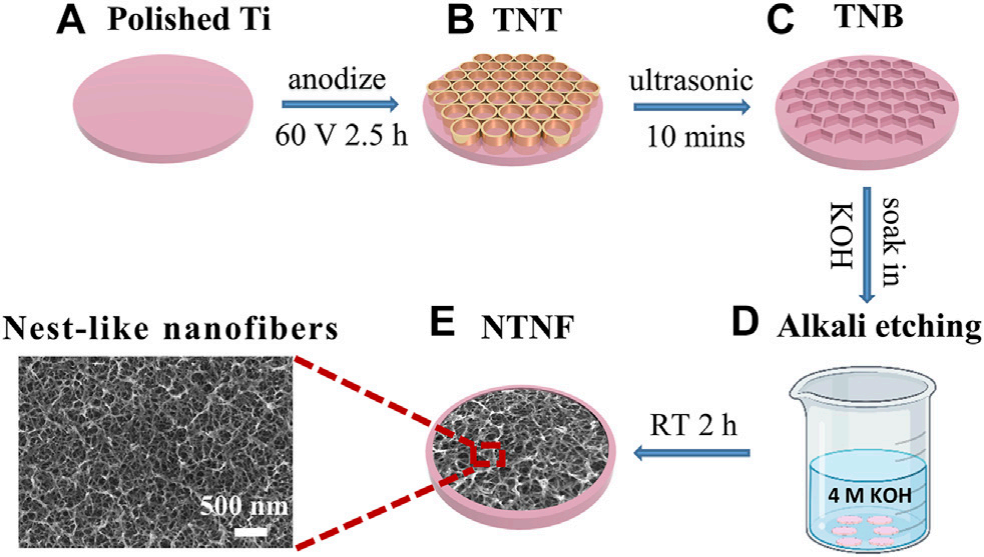
Schematic diagram of preparing TNT and NTNF. **(A)** Polished Ti and **(B)** TiO_2_ nanotubes (TNT) prepared on the surface of titanium by electrochemical anodization at 60 V voltage for 2.5 h; **(C)** titanium nanobowl (TNB) created by sonicating the TNT in deionized water for 10 min to remove the nanotubes **(D)** by soaking TNB in 4M KOH alkaline aqueous solution for 2 h at room temperature and normal pressure for alkaline etching, and **(E)** nest-like nanofiber structure (NTNF).

Inspired by this, a method that can significantly reduce high temperature, high pressure, and strict conditions required for alkali etching was developed. This method involves several steps: first, forming a layer of TiO_2_ nanotubes (TNT) (Figure 1B) on the surface of pure titanium (Figure 1A) by electrochemical anodization; second, removing the TiO_2_ nanotubes after anodization to obtain a highly uniform nanobowl structure (TNB) (Figure 1C) template; and third, immersing the material in an alkaline aqueous solution for 2 h at room temperature and normal pressure (Figure 1D) to form the nest-like titanite nanofiber structure (NTNF) (Figure 1E). We hypothesized that this nest-like titanite nanofiber structures based on the top-down approach promotes osseointegration in the same manner as similar structures prepared by the hydrothermal method. Furthermore, the *in vitro* biocompatibility of rat bone marrow mesenchymal stem cells (rBMSCs) and the *in vivo* bone implant osseointegration of the TNT and NTNF groups in toothless beagles were evaluated.

## 2 Materials and Methods

### 2.1 Materials

Pure titanium discs (**>**99.9%, 12 mm in diameter, 0.2 mm in thickness) and rods (3 mm in diameter) were purchased from Baoji Yuanda Metal Materials Co., Ltd. (China). Ammonium fluoride and ethylene glycol were obtained from Aladdin Biochemical Technology Co., Ltd. (China). Potassium hydroxide was acquired from Chron Chemicals Co., Ltd. (China).

### 2.2 Sample Fabrication

#### 2.2.1 Fabrication of TiO_2_ Nanotube Arrays

Pure Ti discs and rods were anodized for the fabrication of nanotube arrays. Before the electrochemical anodization process, metallographic sandpaper (from 800 # to 7,000 #) was applied to the polished surface, followed by ultrasound with acetone, ethanol, and deionized water. To form TiO_2_ nanotube arrays, pure Ti was anodized in ethylene glycol containing 88 mmol/L ammonium fluoride as the electrolyte at 60 V for 2.5 h at room temperature. These prepared samples were denoted as “TNT” (Supplementary Figure S1A).

#### 2.2.2 Fabrication of Nest-Like Nanofiber Structures

To remove the TiO_2_ nanotube arrays formed, TNT samples were processed in deionized water 10 min using ultrasonic concussion. Regularly arranged nanobowl shapes were obtained on the Ti foil and rod. The nanobowl samples were soaked in 4 mol/L KOH solution for 2 h at room temperature and pressure and then soaked in deionized water for 2 h before ultrasonic shock cleaning. These prepared samples were denoted as “NTNF” (Supplementary Figure S1B).

### 2.3 Sample Characterization

The surface morphologies and distribution of element of the TNT and NTNF samples were analyzed by scanning electron microscopy (SEM, Hitachi S-4800, Japan) and using an energy-dispersive spectrometer (EDS, Oxford X-max80, United States). The surface morphology and roughness of TNT and NTNF were observed by atomic force microscopy (AFM, NX10, Park SYSTEMS, Korea). The chemical composition and phase of the specimens were measured by X-ray diffraction (XRD, PANalytical X’Pert PRO, Netherlands), at a test range of 10°–90°. The chemical compositions and states of the sample surfaces were studied by X-ray photoelectron spectrometry (XPS, Thermo ESCALAB 250, United States).

### 2.4 Biocompatibility Experiments *In Vitro*

#### 2.4.1 Culture of Rat Bone Marrow Mesenchymal Stem Cells (rBMSCs)

The cellular and animal study protocols were approved by the Animal Welfare Ethics Committee of Anhui Medical University. Primary rBMSCs were isolated from the tibiae and femurs of 4-week-old Sprague–Dawley (SD) male rats following a previously described method (Wang et al., 2016). In brief, the bilateral tibiae and femurs of rats were harvested under aseptic conditions, opened at both ends, and flushed with culture media. The isolated cells were suspended in complete α-MEM (*α*-MEM supplemented with 10% FBS and 100 U/ml penicillin/streptomycin), and rBMSCs from passages three to four were used for further *in vitro* experiments. No osteoinductive factors were used in this study.

#### 2.4.2 Early Adhesion and Morphology of rBMSCs on TiO_2_ Nanotubes and Nest-Like Titanite Nanofiber Structures

TNT and NTNF were randomly chosen and placed in 24-well plates. rBMSCs were seeded on the surface of the TNT and NTNF samples and incubated for 1 day. Then, the samples were carefully washed with PBS, followed by fixation with 2.5% glutaraldehyde for 12 h. The surface was then air-dried and coated with gold and observed by SEM. The length of the longest pseudopodia of the rBMSCs was calculated according to the SEM images. If there were multiple cells in one SEM image, the whole cell with the longest pseudopodium was selected. Additionally, confocal laser scanning microscopy (CLSM, Leica TCS-SP5, Germany) was also used to determine the adhesion and morphology of rBMSCs on the TNT and NTNF samples. The samples were carefully washed with PBS, followed by 4% paraformaldehyde fixation for 20 min. The cytoskeleton and cellular nucleus were stained with TRITC phalloidin (Solarbio, China) and DAPI (Beyotime, China) in the dark and then inspected utilizing CLSM.

#### 2.4.3 Viability and Proliferation of rBMSCs on TiO_2_ Nanotubes and Nest-Like Titanite Nanofiber Structures

rBMSCs were seeded on the TNT and NTNF sample surfaces and cultured for 1, 4, and 7 days. The seeding density was 1×10^4^/cm^2^. At each time point, rBMSCs were stained using the LIVE/DEAD^TM^ *Bac*Light^TM^ Bacterial Viability Kit (L7012, Invitrogen, United States), following manufacturer’s instructions, and observed by CLSM to identify whether the cells were alive or dead. rBMSCs were seeded on the TNT and NTNF sample surfaces with the same seeding density and cultured for 1, 3, and 5 days. At each time point, the cell proliferation of rBMSCs was evaluated by the Cell Counting Kit-8 (CCK-8, Dojindo Molecular Technology, Japan), according to the manufacturer’s instructions. Then, the OD value was tested at 450 nm by using a microplate reader (Epoth, BioTek, United States).

#### 2.4.4 Alkaline Phosphatase Staining and Activity of rBMSCs on TiO_2_ Nanotubes and Nest-Like Titanite Nanofiber Structures

BMSCs were seeded on TNT and NTNF surfaces and incubated for 4 and 7 days. At each time point, the samples were washed with PBS carefully, followed by 4% paraformaldehyde fixation for 20 min, stained using the BCIP/NBT Alkaline Phosphatase Color Development Kit (Beyotime, China) according to the protocol and observed by a stereo microscope (SZ61, Olympus, Japan). For the quantitative evaluation of ALP activity, the samples were rinsed with PBS and then lysed with 1% Triton X-100 for 30 min. An Alkaline Phosphatase Assay Kit (Beyotime, China) and a BCA Protein Assay Kit (Beyotime, China) were used to quantify the ALP activity and total protein concentration, respectively.

#### 2.4.5 Real-Time Quantitative PCR (qRT-PCR) Analysis of rBMSCs on TiO_2_ Nanotubes and Nest-Like Titanite Nanofiber Structures

After incubation for 7 and 14 days, qRT-PCR assays were performed to quantitatively assess the expression levels of osteogenic genes (*COL1*, *ALP*, *BMP2*, and *RUNX2*) in rBMSCs. Total RNA extraction was performed by using an Axygen RNA Miniprep Kit (Axygen, Union City, CA, United States), according to manufacturer’s instructions. Reverse transcription was completed by utilizing the Prime Script RT reagent Kit. Then, a real-time PCR assay was performed on an ABI 7500 Sequencing Detection System (Applied Biosystems, Foster City, CA) using SYBR® Premix Ex Taq™ II according to our previous report (Chen et al., 2021). ACTB was denoted as the housekeeping gene. The primer sequences are listed in Table 1.

**TABLE 1:** Primer sequences used for qPCR measurements.

Gene	Primer sequence (F, forward; R, reverse)	Accession number
*ACTB*	F: CCTCTATGACAACACAGT	NM_031144.3
	R: AGCCACCAATCCACACAG	
*COL-1*	F: AGC​TCG​ATA​CAC​AAT​GGC​CT	NM_053304.1
	R: CCT​ATG​ACT​TCT​GCG​TCT​GG	
*ALP*	F: TCA​CTT​CCG​CCC​GGA​ACC​CT	NM_013059.2
	R: TGT​CCT​GCC​GGC​CCA​AGA​GA	
*BMP2*	F: GCA​TGT​TTG​GCC​TGA​AGC​AG	NM_017178.2
	R: CGA​TGG​CTT​CTT​CGT​GAT​GG	
*RUNX2*	F: ATC​ATT​CAG​TGA​CAC​CAC​CA	NM_001278483.1
	R: GTA​GGG​GCT​AAA​GGC​AAA​AG	

### 2.5 *In Vivo* Evaluation of Bone Implant Osseointegration

#### 2.5.1 Bone Implant Osseointegration Beagle Model

As shown in Figure 5A, premolars and first molars of beagle dogs were removed under general anesthesia. At 3 months after tooth extraction, titanium rods (3 mm in diameter) were implanted in the alveolar bone of the TNT and NTNF groups. Calcein (20 mg/kg, Solarbio, China) and alizarin red (30 mg/kg, Solarbio, China) were injected into the beagles for double-fluorescence labeling at 9 and 11 weeks after implantation. When osseointegration was completed 3 months later, all beagle dogs were euthanized. The jaws with titanium rod implants were excised and fixed with 4% paraformaldehyde for 48 h and then soaked in 75° alcohol after rinsing overnight with running water.

#### 2.5.2 Micro-CT Measurement and Fluorescent Labeling

The titanium rod specimens were inspected by micro-CT (μCT100, Scanco, Switzerland), and system software was used for three-dimensional reconstruction and quantitative analysis, including bone volume per tissue volume (BV/TV), trabecular thickness (Tb.Th), bone mineral density (BMD), and bone surface/volume ratio (BS/BV) (Zhang et al., 2022). Double-fluorescence labeling was used to evaluate the osteointegration between the bone and implant. The samples were cut into sections with a thickness of 150 μm using a hard tissue slicer (310, EXAKT, Germany), and the cross profiles were polished to a thickness of approximately 40 μm. The mineralization rate was measured by dividing the distance of two fluorescence signals per day.

### 2.6 Statistical Analysis

All values are presented as mean ± standard deviation (SD). Differences between the TNT and NTNF groups were evaluated using Student’s t-test with GraphPad Prism 8.0 software. Significance was determined at *p < 0.05 and **p < 0.01.

## 3 Results and Discussion

### 3.1 Sample Fabrication and Characterization

**FIGURE 2:**
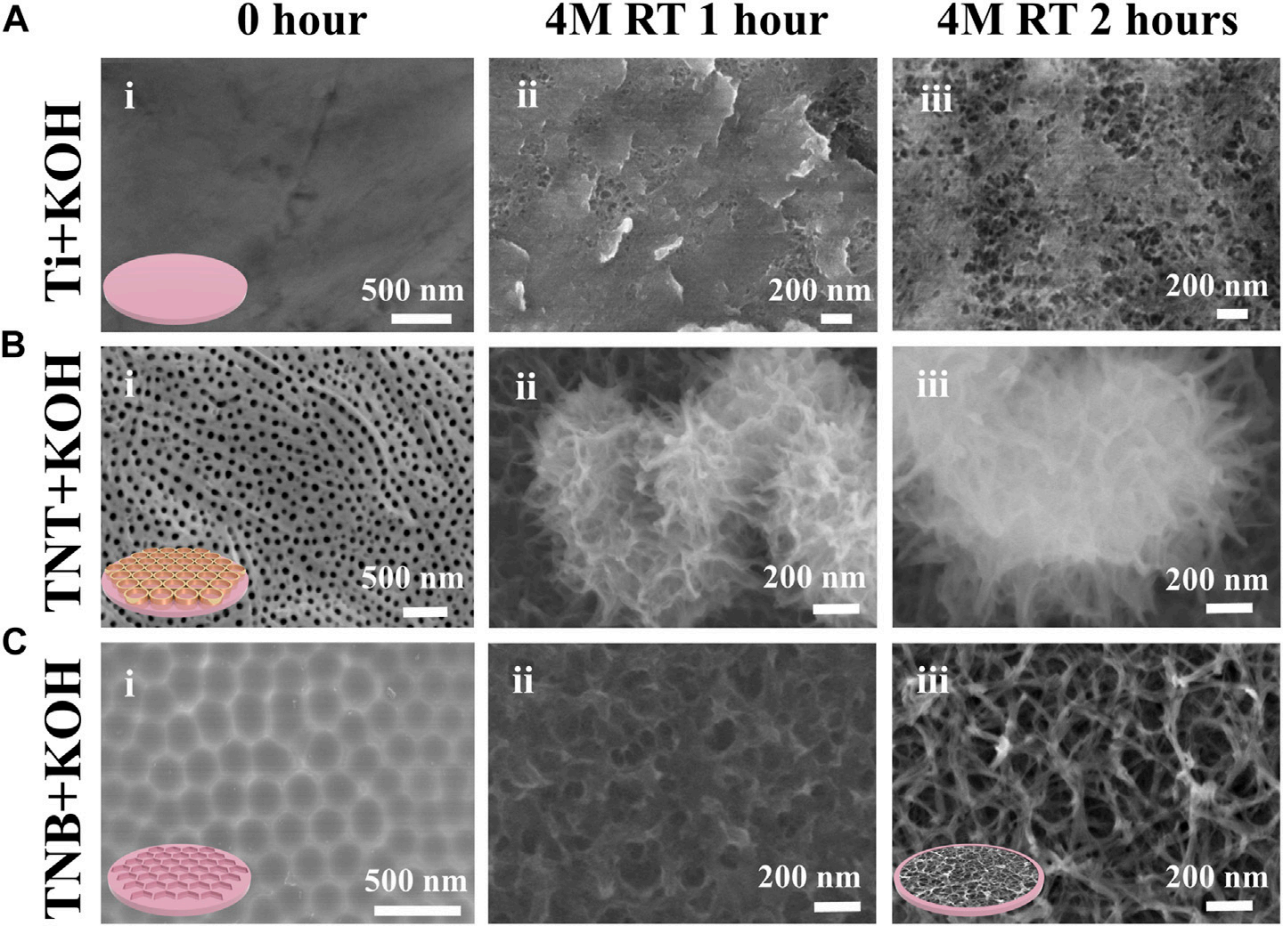
FE-SEM images of **(A)** Ti, **(B)** TNT, and **(C)** TNB were soaked in 4M KOH alkaline aqueous solution under normal temperature and pressure for 0, 1, and 2 h.

**FIGURE 3:**
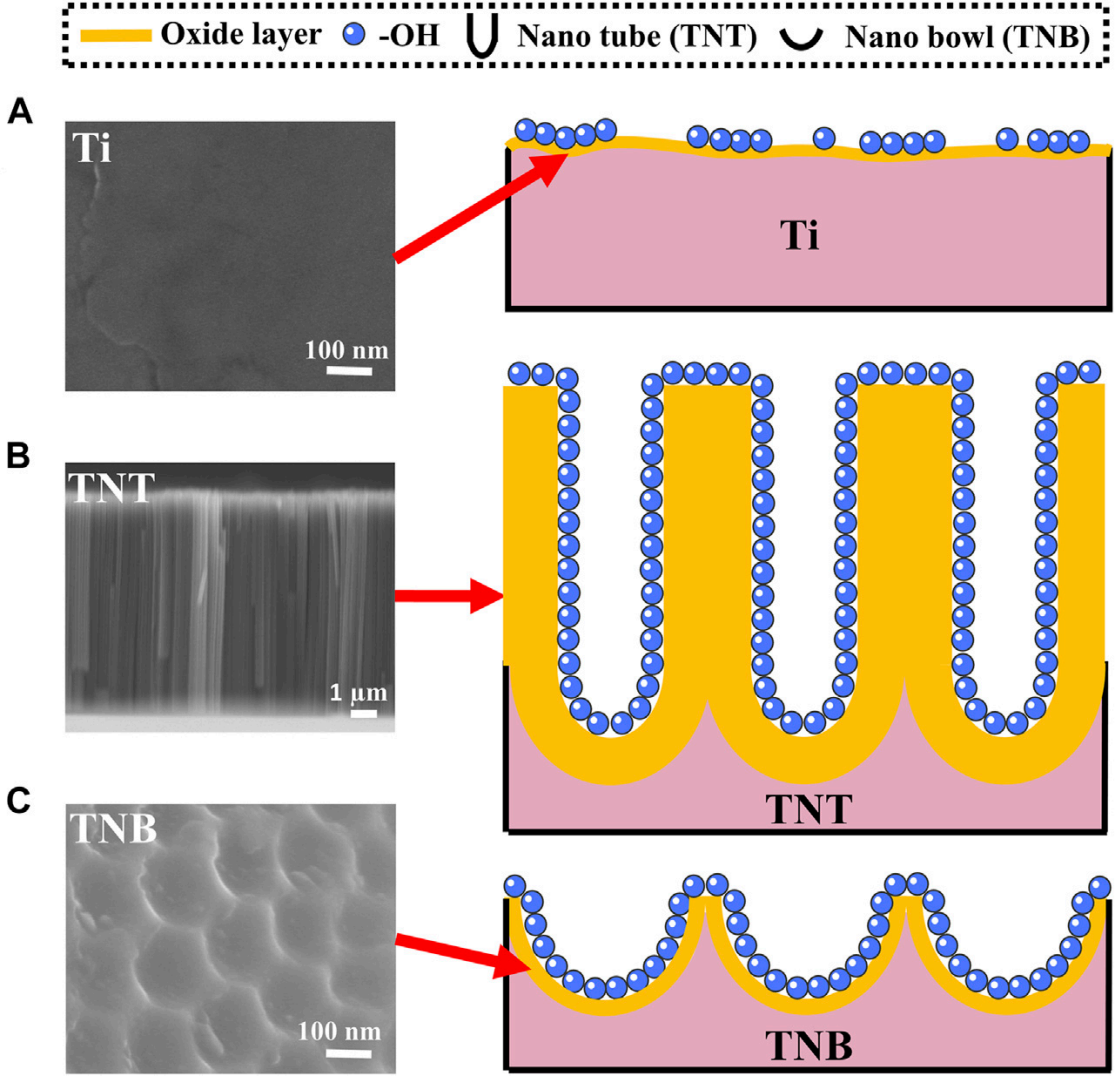
Schematic diagram of surface etching of KOH alkaline solution on **(A)** Ti, **(B)** TNT, and **(C)** TNB.

Dents and scratches can be seen on the surface of pure titanium in Figure 2 and Figure 3A. A TiO_2_ nanotube array (TNT) with a pore size of approximately 70–80 nm and a thickness of approximately 7–8 μm was formed on the surface of pure titanium after electrochemical anodization at 60 V for 2.5 h (Figures 2Bi, 3B). After the nanotube array was shaken off by ultrasonic vibration, 5–6 polygonal honeycomb-like uniformly arranged nanobowls (TNB) were left on the surface of pure titanium (Figures 2Ci, 3C). Figure 2 shows SEM images of the surfaces of the Ti, TNT, and TNB substrates treated with 4M KOH solution at room temperature and pressure for 0 h, 1 h, and 2 h, respectively. After 1 h of alkali etching, the surface of Ti changed to uneven status from a relatively flat surface, and the corroded nano-holes can be seen in some areas (Figure 2Aii). TNT is corroded and collapsed into loose three-dimensional grid-like nanospheres that are easily exfoliated and attached to the titanium surface (Figure 2Bii). TNB is etched as mutually connected holes (Figure 2Cii). After 2 h alkali etching, the number of nanoholes corroded on the Ti surface increases, but they are not completely connected with each other (Figure 2Aiii). The nanospheres formed after TNT etching are further corroded and fused into large and loose three-dimensional grid-like nanospheres (Figure 2Biii). The surface of TNB is further etched into the nested nanofiber structure with connected holes (Figure 2Ciii). The same TNT and NTNF structures can also be seen on the titanium rods (Supplementary Figure S3).

A thin (1.5–10 nm) oxide layer is formed on the surface of the titanium when exposed to the air (Lee et al., 2019; Gianfreda et al., 2021). At the same time, there are slight dents and scratches on the surface (Wang et al., 2019), which leads to uneven and insufficient contact of -OH in the alkaline solution and pure titanium during alkaline etching (Figure 3A). Because etching efficiency is positively correlated with the concentration of -OH, a certain concentration of alkali solution can only partially etch the surface into discontinuous holes. TNT is a TiO_2_ nanotube prepared by anodic oxidation (Sun et al., 2018). The inside and wall of the tube are fully contacted with -OH (Figure 3B). Although it can be sufficiently etched by alkali, it could cause the nanotubes to collapse and adhere to the titanium surface, and the adhesion is weak and easy to detach. On the other hand, the TNB has a regular and uniform nanostructure on the titanium surface and an increased specific surface area. The oxide layer is also thickened, so that the -OH in the alkaline solution can fully and uniformly contact the titanium surface (Figure 3C), which robustly increases chemical reaction efficiency (Hou et al., 2018; Hamans et al., 2020; Zhang et al., 2020) and can create nest-like nanofiber structures by soaking in alkaline solution for 2 h under normal temperature and pressure. It is also worth mentioning that the scratch test (Supplementary Figure S3) results show that the SEM images of the NTNF surface do not show the peeling layer after being scratched with the tip of the tweezers compared to that of TNT, indicating that the NTNF structure prepared by top-down etching under normal temperature and pressure has higher adhesion and stability.

**FIGURE 4:**
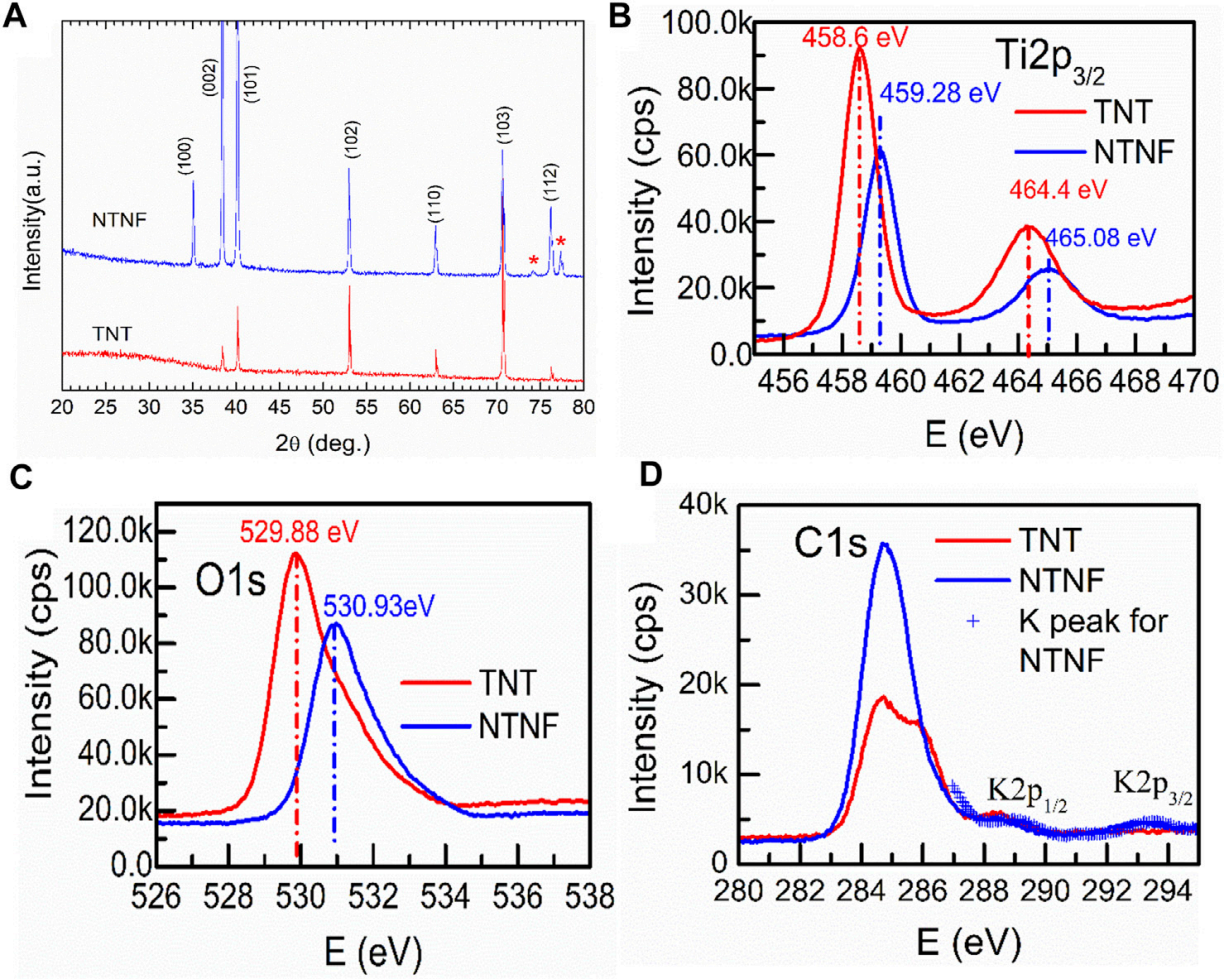
**(A)**XRD patterns of the TNT and NTNF. The XPS patterns around Ti_2*p*_ **(B)**, O_1*s*_ **(C)**, C_1*s*_, and K_2*p*_ **(D)** peaks for the TNT and NTNF samples.

Figure 4A shows the XRD patterns for the TNT and NTNF samples. The majority phase for both samples can be indexed to pure Ti (PDF05-0682). Since the top surface of the TNT sample is covered by amorphous TiO_2_ nanotubes, the intensity of the XRD peaks of TNT is lower. In addition, amorphous TiO_2_ nanotubes do not show any additional peaks. When TNB is immersed in KOH aqueous solution, the oxide layer on the surface is partially dissolved due to the corrosion of hydroxyl (-OH). At the same time, titanium reacts with alkaline solution through hydration reaction, and it further attacks on the hydroxyl group of hydrated titanium dioxide (TiO_2_.nH_2_O) to produce negatively charged hydrates ([HTiO_3_¯]. nH_2_O) (Shin et al., 2016). Therefore, the formed titanite (red asterisk) can be observed on the XRD pattern of NTNF (Raveendra et al., 2018). Supplementary Figure S4 shows the full-scale XPS patterns for the TNT and NTNF samples. All the major peaks corresponding to Ti_3*p*_, Ti_3*s*_, Ti_2*p*_, Ti_2*s*_, C_1*s*_, O_1*s*_, and F_1*s*_ are labeled. Generally, except for the F_1*s*_ peak, the XPS pattern changes little after KOH application. The F_1_ peak for the TNT sample indicates that F ions are introduced during the electrochemical process in NH_4_F solution. After applying the KOH aqueous solution, F ions are removed. Figure 4B shows the XPS patterns around the Ti_2*p*_ peaks for the TNT and NTNF samples. As is shown, both samples show two combining energy peaks for Ti_2*p*3/2_ and Ti_2*p*1/2_. For the TNT sample, the Ti_2*p*3/2_ and Ti_2*p*1/2_ peak positions are 458.6 and 464.4 eV, respectively, which is a signature of Ti^4+^ ions (Yang et al., 2005). After applying KOH, the peak positions of Ti_2*p*_ for NTNF sample shift to higher energy positions, namely, from 458.6 eV Ti_2*p*3/2_ and 464.4 eV Ti_2*p*1/2_ for the TNT sample to 459.28 eV Ti_2*p*3/2_ and 465.08 eV Ti_2*p*1/2_ for the NTNF sample. This finding demonstrates that oxidation state of Ti on the surface of NTNF sample is not +4. Figure 4C shows the XPS patterns around O_1*s*_ peaks for the TNT and NTNF samples. The combining energy ∼529.88 eV for the TNT sample is a typical value for lattice-O, while ∼531 eV for the NTNF sample is a typical value for absorbed O_2_ (Yang et al., 2006). Generally speaking, the Ti-O layer on the TNT sample surface was removed by KOH dealing. As shown in Figure 4D, the XPS pattern around K_2*p*_ peak for NTNF sample indicates the existence of K ions on the surface of NTNF sample. Generally, the existence of K ions and the shift of Ti_2*p*_ peaks from XPS for NTNF sample confirm that the new peaks from XRD pattern should be titanite.

**FIGURE 5:**
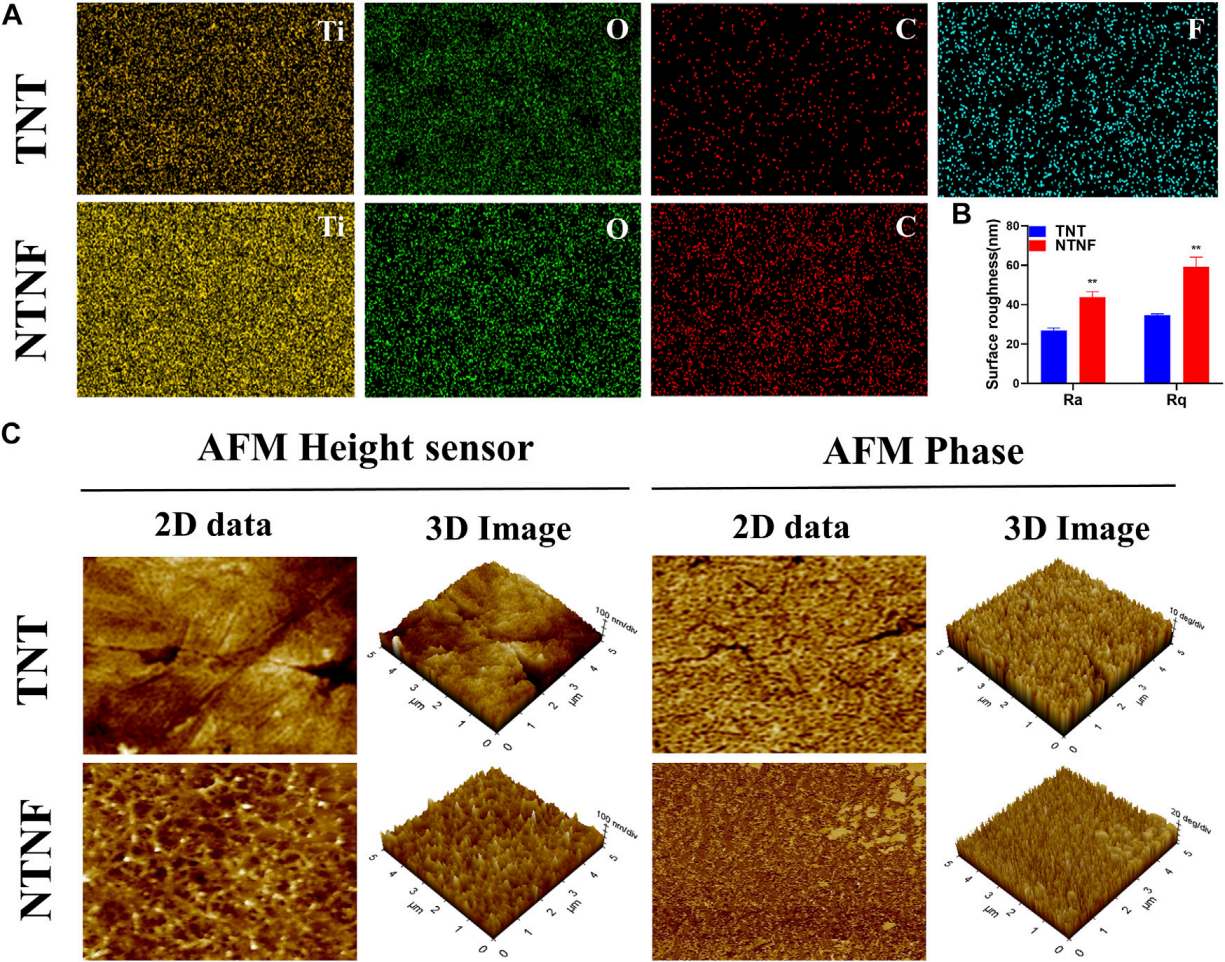
**(A)** EDS mapping images of the TNT and NTNF. **(B)** Surface roughness measured by AFM. Ra, arithmetic average roughness; Rq, root-mean-square roughness. **p < 0.01 compared with the TNT group. **(C)** 2D and 3D AFM images of height sensor, phase, and amplitude on the surface of the TNT and NTNF.

EDS analyzed the chemical composition of the TNT and NTNF samples (Supplementary Figure S5; Supplementary Table S1). Figure 5A and Supplementary Figure S6 show the EDS mapping images of the TNT and NTNF [Ti (yellow), O (green), C (red), and F (blue)]. Apparently, the oxygen ratio for the NTNF sample is smaller than that of the TNT sample. The surface roughness of the implants influences cellular adhesion, dispersal, and proliferation *in vitro* and *in vivo* (Zhang et al., 2018; Bernhardt et al., 2021). The surface roughness of the materials refers to the unevenness of the surface with small peaks and valleys, which is correlated with surface smoothness, a critical factor for cell adhesion and proliferation (Zhou et al., 2021). Ra, the arithmetic average roughness, is a commonly used parameter of roughness. Rq is the root-mean-square roughness, which is the root-mean-square value of the deviation between the contour and the average line. For the TNT group, the Ra value was 26.82 ± 1.32 nm and the Rq value was 34.68 ± 0.70 nm, and for the NTNF group, the Ra value was 43.80 ± 2.78 nm and the Rq value was 59.29 ± 4.80 nm, which was significantly greater than that of the TNT group (Figure 5B, *p* < 0.01). The AFM topography and phase images of NTNF and TNT are shown in Figure 5C. Cracks between nanotube arrays could be seen on the surface of TNT. The surface of the NTNF group had a rougher appearance than that of the TNT group, which is consistent with the SEM results.

### 3.2 Effects of TiO_2_ Nanotubes and Nest-Like Titanite Nanofiber Structures on the Adhesion and Morphology of rBMSCs

**FIGURE 6:**
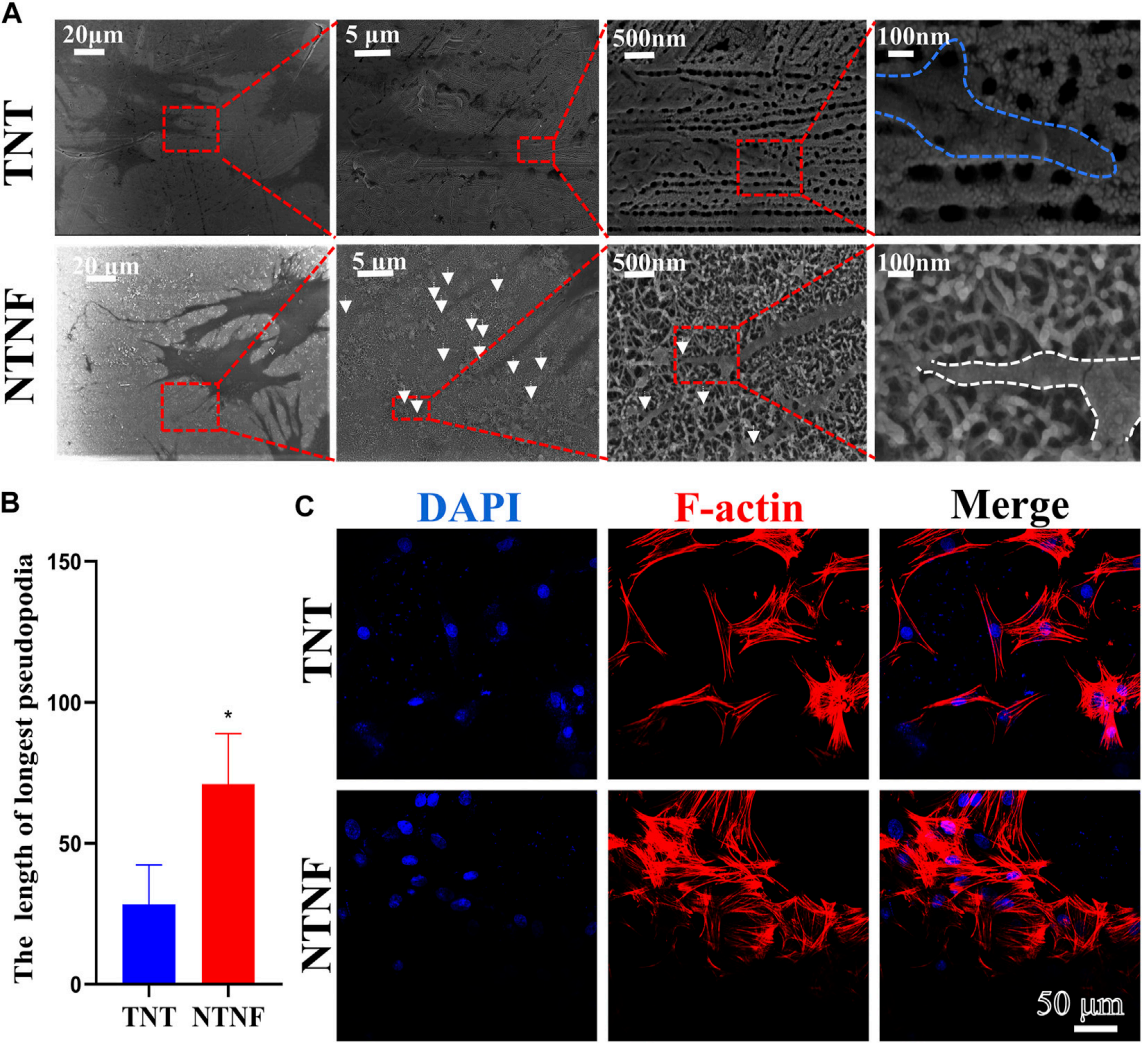
**(A)** SEM images of rBMSCs attached on the surface of TNT and NTNF for 24 h. White arrows refer to the tentacles of rBMSC. **(B)** Length of filamentous pseudopodia of the rBMSCs on the TNT and NTNF samples surface. **(C)** CLSM images of rBMSCs morphology and attachment after culturing on TNT and NTNF for 24 h by immunofluorescence staining [nuclei (blue), f-actin (red)].

Next, the adhesion and morphology of rBMSCs on the surface of TNT and NTNF were evaluated by SEM after 1 day of cell culture. As shown in Figure 6A, the SEM images showed that a large number of slender filamentous pseudopodia protruded from the rBMSCs on the NTNF surface, and these pseudopodia were marked by white arrows. Intriguingly, it was observed that the ends of the pseudopodia all protruded into the grid structure of the nest-like nanofiber structure (white dotted line). However, the filamentous pseudopodia of the rBMSCs on the TNT surfaces were obviously fewer and shorter, and the ends of the pseudopodia were spread flat in the vicinity of the nozzle of TiO_2_ nanotubes (blue dotted line). The pseudopod of cell is related to cytoskeleton remodeling and cell adhesion, which is critical for cell spreading and cell migration (Jia et al., 2005). The length of filamentous pseudopodia of the rBMSCs on the NTNF surface was longer than that of the TNT surface (*p* < 0.05, Figure 6B). To further evaluate the morphology of rBMSCs on the TNT and NTNF surfaces, the cytoskeleton was stained with phalloidin for immunofluorescence detection by CLSM. As shown in Figure 6C, the number of rBMSCs on the surface of the NTNF samples increased slightly and was arranged more tightly, with extensively stretched morphologies, compared with that on the TNT samples. Moreover, the pseudopodia of the rBMSCs on the surface of the NTNF samples were interlaced with each other. These results indicated that NTNF effectively promoted the early adhesion and dispersed morphology of rBMSCs compared with TNT, which may be attributed to the nest-like nanofiber structure of NTNF.

### 3.3 Effects of TiO_2_ Nanotubes and Nest-Like Titanite Nanofiber Structures on Cell Viability and Proliferation

**FIGURE 7:**
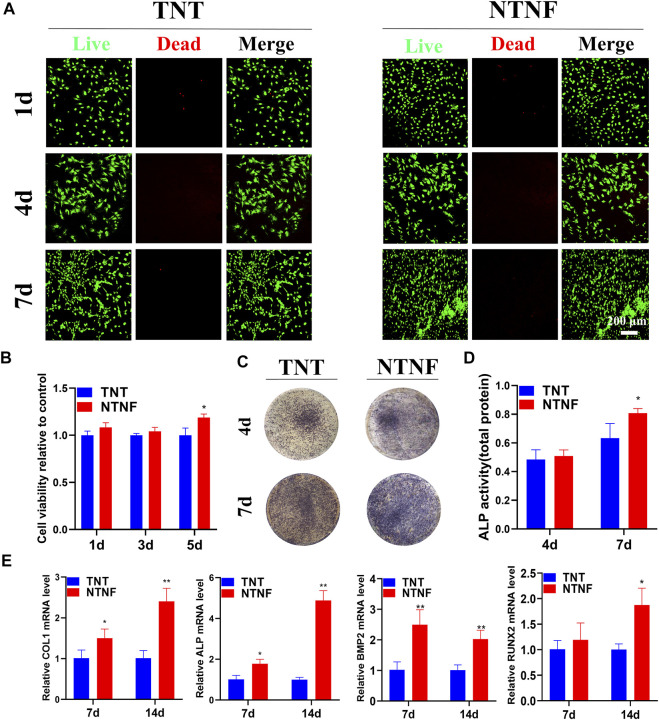
**(A)** Live/dead (SYTO 9/PI) staining images of rBMSCs cultured on the surface of TNT and NTNF for 1, 4, and 7 days. **(B)** Cell counting kit-8 (CCK-8) assay of rBMSCs cultured on TNT and NTNF. **(C)** Alkaline phosphatase (ALP) staining and **(D)** activity images of rBMSCs in the different groups after 4 and 7 days. **(E)** Expression of osteogenesis-related genes analyzed by RT-qPCR in rBMSCs cultured on different samples for 7 and 14 days. *p < 0.05, **p < 0.01 compared with the TNT group.

The cell viability of rBMSCs on the surface of the TNT and NTNF samples was evaluated by LIVE/DEAD staining on days 1, 4, and 7. As shown in Figure 7A, the green-stained live rBMSCs exhibited a normal morphology and adhered to the surface of all samples, in which few red-stained dead rBMSCs were found. As the cultivation time increased, the number of rBMSCs on the surface of the TNT and NTNF increased. To evaluate the cell viability and proliferation rate with a more quantitative approach, a CCK-8 assay was performed on days 1, 3, and 5 (Figure 7B). The number of rBMSCs on the surface of NTNF gradually increased during the culturing period, and obvious cytotoxicity was not observed compared with TNT samples. Although no prominent difference in proliferation rate was observed between the TNT and NTNF samples on days 1 and 3, a higher cell proliferation of rBMSCs on the surface of NTNF was observed on day 5, compared with TNT (*p* < 0.05). These results showed that rBMSCs exhibited satisfactory cell viability and proliferative performance on the surface of NTNF.

### 3.4 Effects of TiO_2_ Nanotubes and Nest-Like Titanite Nanofiber Structures on Alkaline Phosphatase Activity

Encouraged by the satisfactory biocompatibility of NTNF, we next explored whether the nest-like nanofiber structure of NTNF promotes the osteogenetic process of rBMSCs. As an early marker of osteogenic differentiation, alkaline phosphatase (ALP) is mainly distributed in the cell membrane as a transporter to promote osteoblast maturation and calcification (Gonzalez Ocampo et al., 2019). The quantitative detection of ALP can reflect the differentiation level of osteoblasts. As shown in Figure 7C, the rBMSCs exhibited more distinct ALP staining after 7 days of culture on the NTNF samples than that on the TNT samples. However, no prominent difference was observed between the TNT and NTNF samples on day 4. To further explore the ALP activity quantitatively, the ALP activity was measured after culturing rBMSCs on the TNT and NTNF samples for 4 and 7 days. The quantitative analysis demonstrated that there was no prominent difference in the ALP activity between the TNT and NTNF groups on day 4. However, the activity level of NTNF was significantly higher than that of TNT on day 7 (Figure 7D, *p* < 0.05). Taken together, these data illustrated that compared with TNT, NTNF exhibited enhanced osteogenic capacity by upregulating the ALP activity.

### 3.5 Effects of TiO_2_ Nanotubes and Nest-Like Titanite Nanofiber Structures on the Expression of Osteogenesis-Related Genes

To further investigate the osteogenesis-inducing capability of NTNF, RT-qPCR was used to quantify the expression of osteoblast-related genes, including *ALP*, *COL1*, *RUNX2*, and *BMP2*. As described in Figure 7E, the NTNF samples significantly enhanced the expression of *COL1*, *ALP*, and *BMP2* at day 7 (*p* < 0.05 for *COL1* and *ALP*, *p* < 0.01 for *BMP2*). Similar results could also be detected at day 14, when the expression of *COL1*, *ALP*, *BMP2*, and *RUNX2* were significantly upregulated in the NTNF group compared with the TNT group (*p* < 0.05 for *RUNX2*, *p* < 0.01 for *COL1*, *ALP*, and *BMP2*). Overall, these results suggested that NTNF promoted highly efficient osteogenic differentiation at the transcriptional level.

### 3.6 Effects of TiO_2_ Nanotubes and Nest-Like Titanite Nanofiber Structures on Osteogenesis and Bone Implant Osseointegration *in vivo*

**FIGURE 8:**
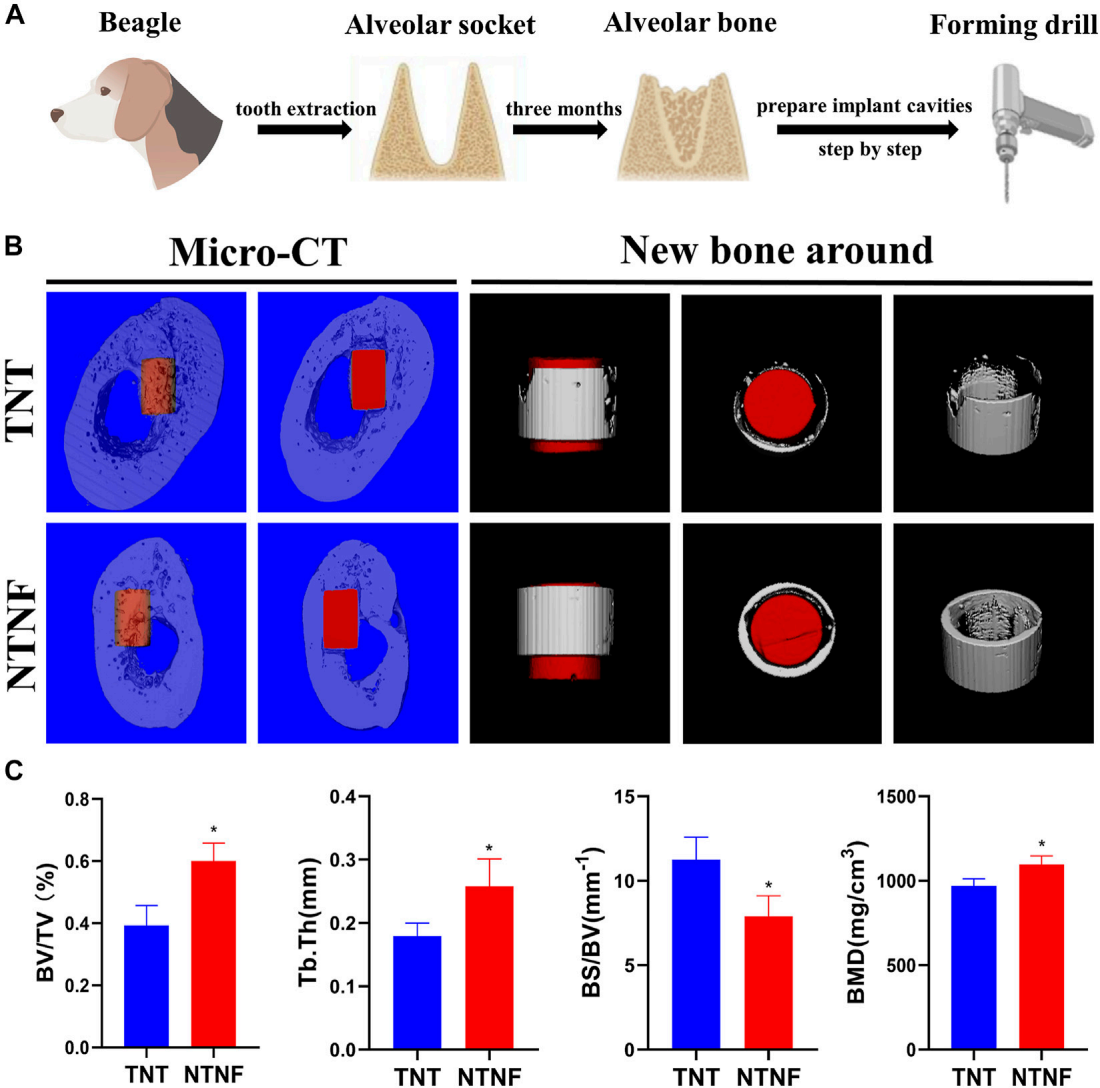
**(A)** Surgical preparation of titanium rod before implantation. **(B)** Application of TNT and NTNF implant in beagle jaw, illustrated as micro-CT images including 3D reconstruction and new bone around of the bone repair 3 months after implantation. **(C)** BV/TV, Tb.Th, BS/BV, and BMD of the bone volume adjacent to the implant surface were quantified, respectively (*n* = 3,*p < 0.05).

After evaluating the *in vitro* biocompatibility and osteogenic performance, the *in vivo* application of NTNF by establishing a beagle alveolar implant model was explored (Figure 8A). The reason for choosing large animals such as beagle is that its jaw is similar to that of humans, which can better recapitulate the osseointegration of implants *in vivo* (Hakanen et al., 2018). Micro-CT and fluorescent labeling analyses were utilized to detect osteointegration after 3 months of implantation (Figure 8B; Supplementary Figures S7, S8). As shown in Figure 8C, the percent bone volume (BV/TV), trabecular thickness (Tb.Th), and bone mineral density (BMD) of the NTNF group were higher than those of the TNT group (*p* < 0.05), while the bone surface/volume ratio (BS/BV) of the NTNF group was lower than that of the TNT group (*p* < 0.05).

**FIGURE 9:**
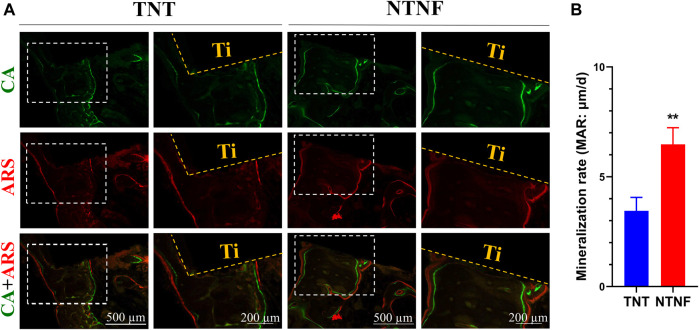
**(A)** Fluorochrome labeling analysis by calcein at 9 weeks and alizarin red at 11 weeks before euthanasia. **(B)** Quantitative analysis of mineralization rates for TNT and NTNF groups (*n* = 3, **p < 0.01).

The new bone tissue was qualitatively assessed employing double-fluorescence labels at 9 and 11 weeks after titanium bar implantation. As shown in Figure 9A, green and red represent calcein (CA) and alizarin red (ARS) injected into the beagle at 9 and 11 weeks, respectively, and the distance between them represents the formation of new bone. The quantification shows that the dividing distance between CA and ARS was significantly higher in the NTNF group than that in the TNT group (Figure 9B, *p* < 0.01). These data indicated that the newly formed bone area around the bone implants in the NTNF group was higher than that in the TNT group, which is consistent with the micro-CT observations. Consistent with the *in vitro* study, the *in vivo* data illustrated that the nest-like nanofiber structure provided a favorable microenvironment for osteoblast adhesion, dispersal, and differentiation, thus contributing to satisfactory bone implant osseointegration in the beagle model.

## 4 Conclusion

In this study, TNT was prepared on the surface of pure titanium through electrochemical anodization, which has a pore size of approximately 70–80 nm and a thickness of approximately 7–8 μm. After ultrasonic concussion to remove TNT, a honeycomb-like uniformly arranged TNB template with about 160 nm diameter was formed to improve the reaction efficiency of alkali etching greatly and prepared the NTNF, which was nest-like nanofiber structure under normal temperature and pressure.

The titanium surface with this nest-like nanofiber structure can promote the adhesion, viability and proliferation, osteogenesis-related gene expression, and osteogenic differentiation of rBMSCs *in vitro* and promote the osteogenesis and osseointegration of the implant beagle model. Therefore, the NTNF structure has higher stability, biocompatibility, and osteogenesis than TNT. Considering the simple, efficient, and safe fabrication of electrochemical anodic oxidation and alkali etching methods, titanium implants modified with the NTNF structure possess high clinical application value.

## Data Availability

The original contributions presented in the study are included in the article/Supplementary Material; further inquiries can be directed to the corresponding authors.
