# Supplementary material for: Titanium Nanobowl-Based Nest-Like Nanofiber Structure Prepared at Room Temperature and Pressure Promotes Osseointegration of Beagle Implants
Source: Front Bioeng Biotechnol. 2022 Feb 24;10:841591. doi: 10.3389/fbioe.2022.841591 (PMC8908903; doi:10.3389/fbioe.2022.841591)
Supplement: Supplementary file 1 [file Presentation1.pdf]

Supplementary Materials for

# Titanium nanobowl-based nest-like nanofiber structure prepared at room temperature and pressure promotes osseointegration of beagle implants

**Lei Sun[1,2], Xuzhuo Chen[3], Haizhang Mu[3], Yin Xu[4], Ruiguo Chen[5], Rong Xia[2]*, Lunguo Xia[6]*, Shanyong Zhang[1,3]***

[1]Department of Oral and Maxillofacial Surgery, School and Hospital of Stomatology, Cheeloo College of Medicine, Shandong University & Shandong Key Laboratory of Oral Tissue Regeneration & Shandong Engineering Laboratory for Dental Materials and Oral Tissue Regeneration, Jinan 250012, Shandong, China

[2]Department of Stomatology, the Second Affiliated Hospital of Anhui Medical University, Hefei 230601, Anhui, China

[3]Department of Oral Surgery, Ninth People's Hospital, College of Stomatology, Shanghai Jiao Tong University School of Medicine; Shanghai Key Laboratory of Stomatology & Shanghai Research Institute of Stomatology, National Clinical Research Center of Stomatology. Shanghai 200011, China

[4]Laboratory of Molecular Neuropsychology, School of Mental Health and Psychological Sciences, Anhui Medical University, Hefei 230032, Anhui, China

[5]High Magnetic Field Laboratory, CAS Key Laboratory of High Magnetic Field and Ion Beam Physical Biology, Hefei Institutes of Physical Science, Chinese Academy of Sciences, Hefei 230031, Anhui, China

[6]Department of Orthodontics, Ninth People's Hospital, Collage of Stomatology, Shanghai Jiao Tong University School of Medicine, Shanghai 200011, China.

* Correspondence: xiarongqh@aliyun.com (R.X.); xialunguo@hotmail.com (L.X.); ZHANGSY1787@sh9hospital.org.cn (S.Z.)

**Keywords:** titanium, nanofiber, biocompatibility, osseointegration, implant

**This PDF file includes:**

Figs. S1 to S8
Table S1

**Figure S1.** Physical specimens of TNT and NTNF.

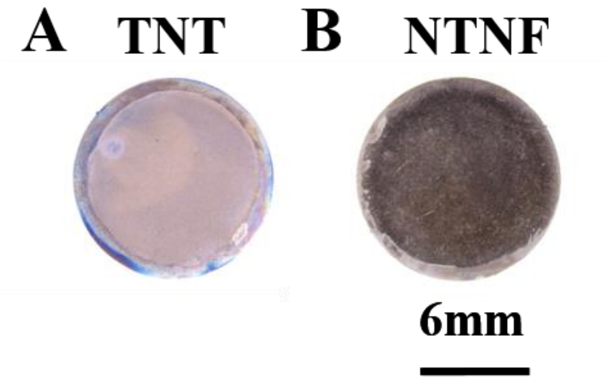

**Figure S2.** The SEM of the titanium rods of TNT and NTNF.

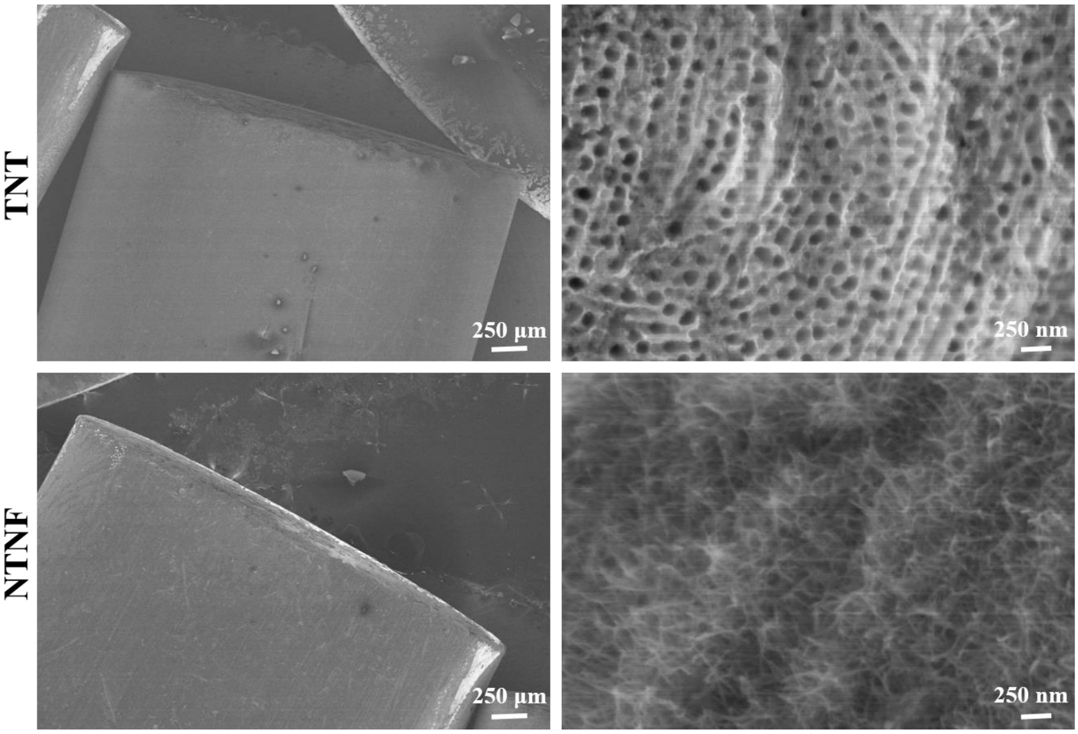

**Figure S3.** Scratch experiment on the surface of TNT and NTNF samples.

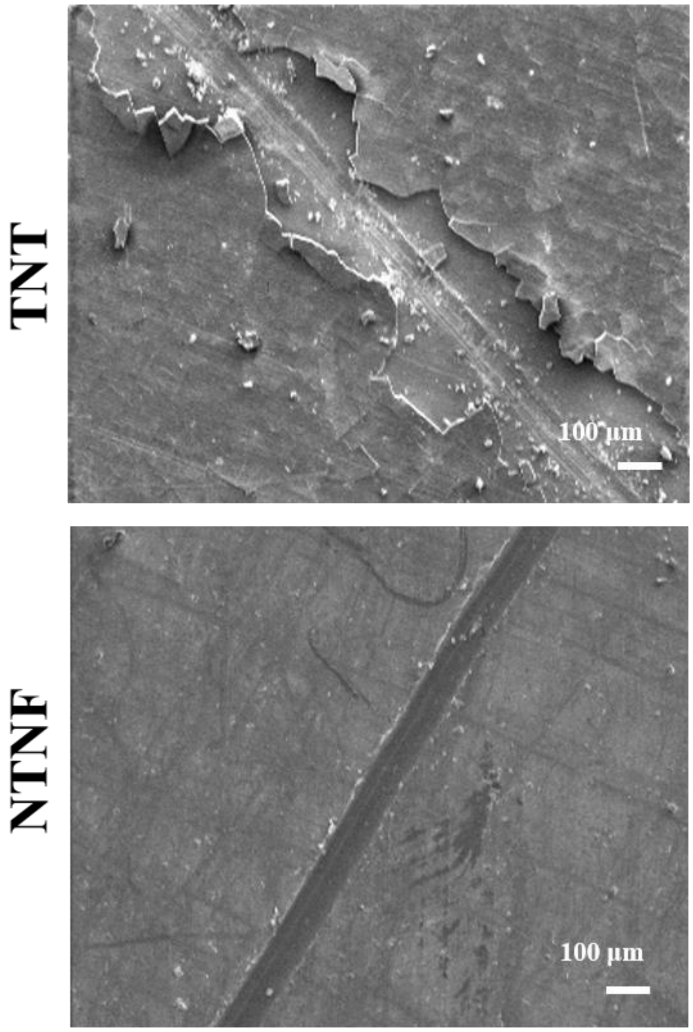

**Figure S4.** The full scale XPS patterns for TNT and NTNF samples.

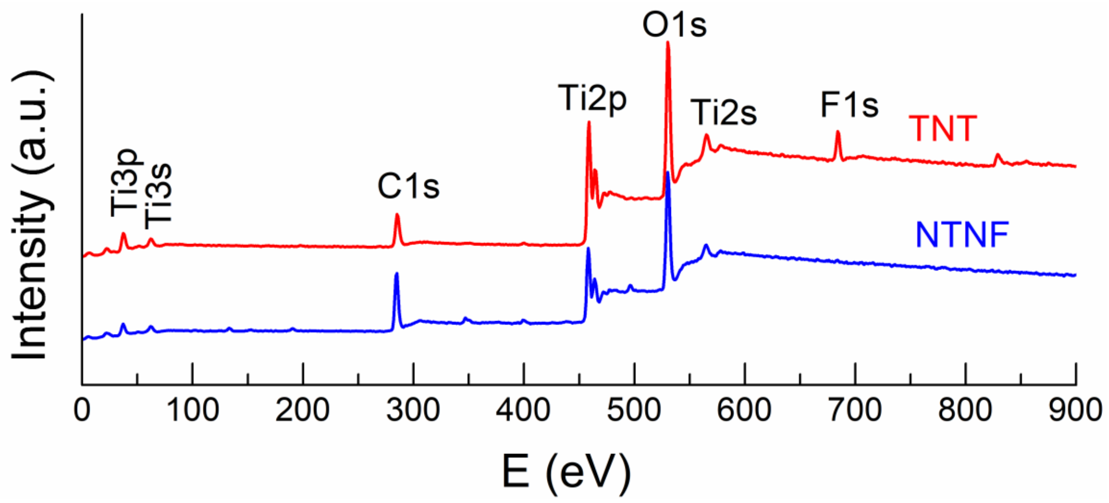

**Figure S5.** The EDS analysis of the (A) TNT and (B) NTNF.

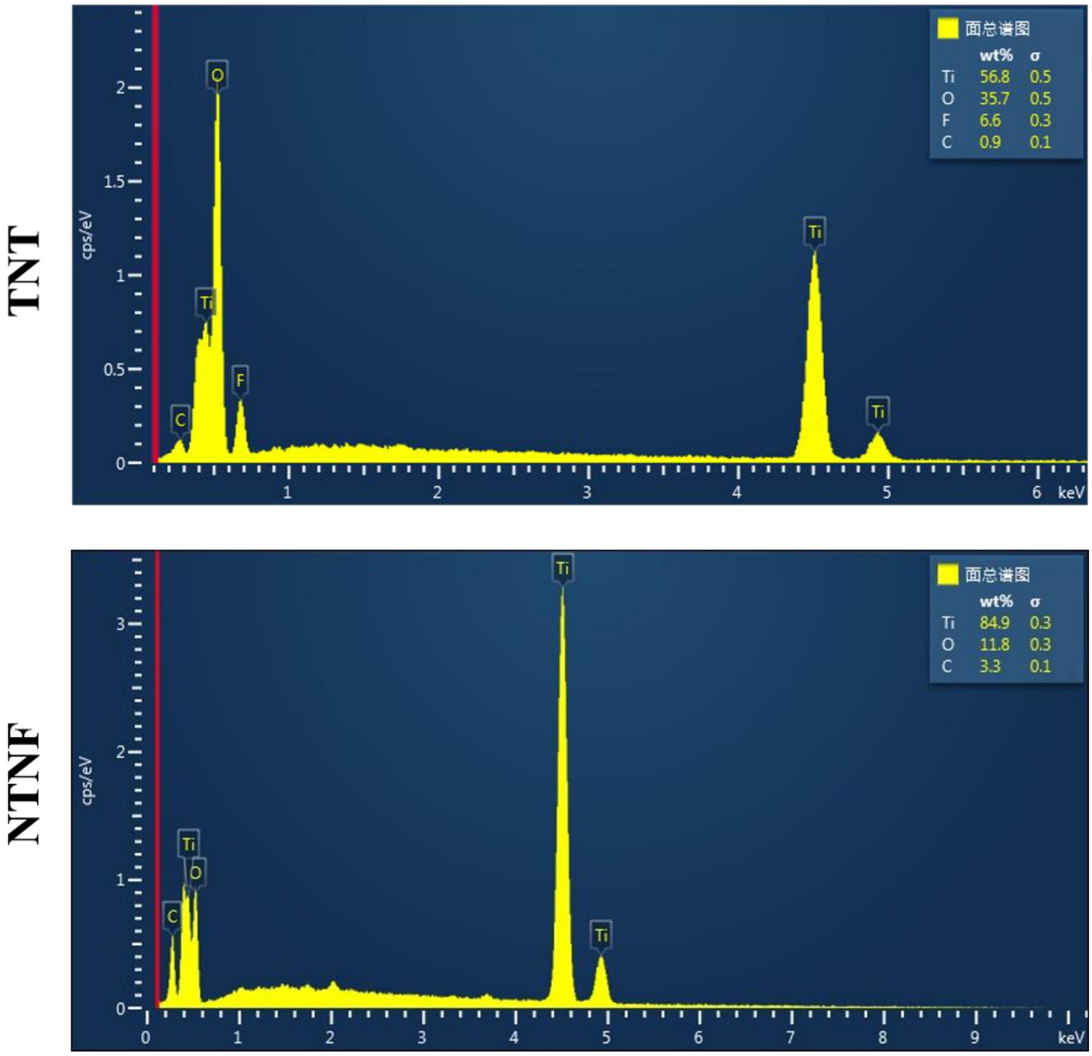

**Figure S6.** EDS mapping images of the TNT and NTNF ( Ti (yellow), O(green), C(red) and F(blue)).

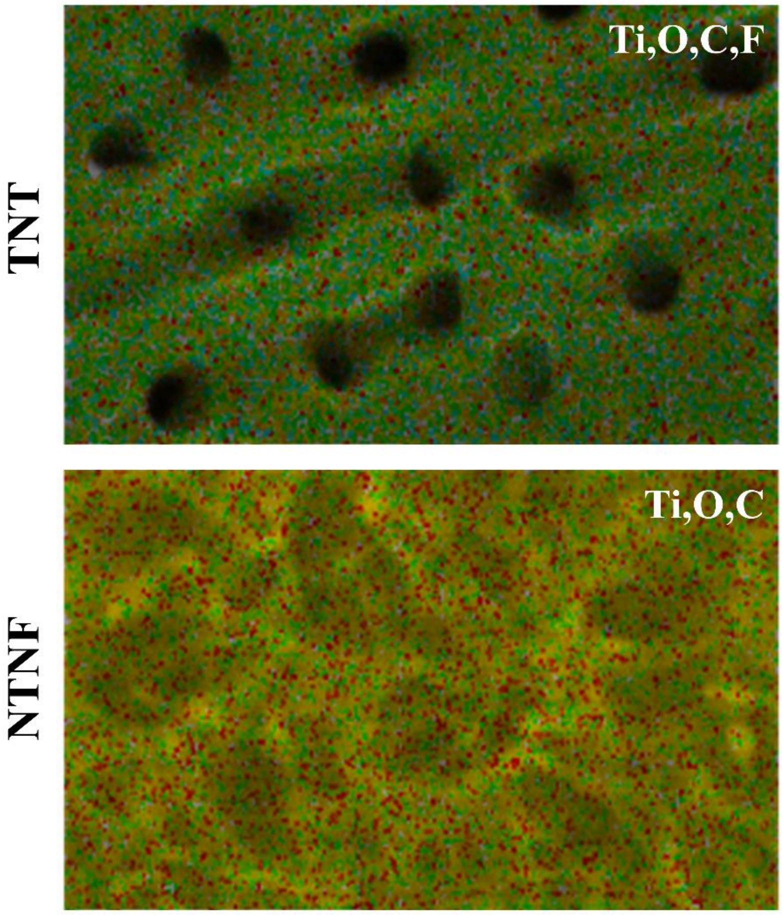

**Figure S7.** Micro-CT 3D reconstruction on beagle jaw at 12 weeks after implantation.

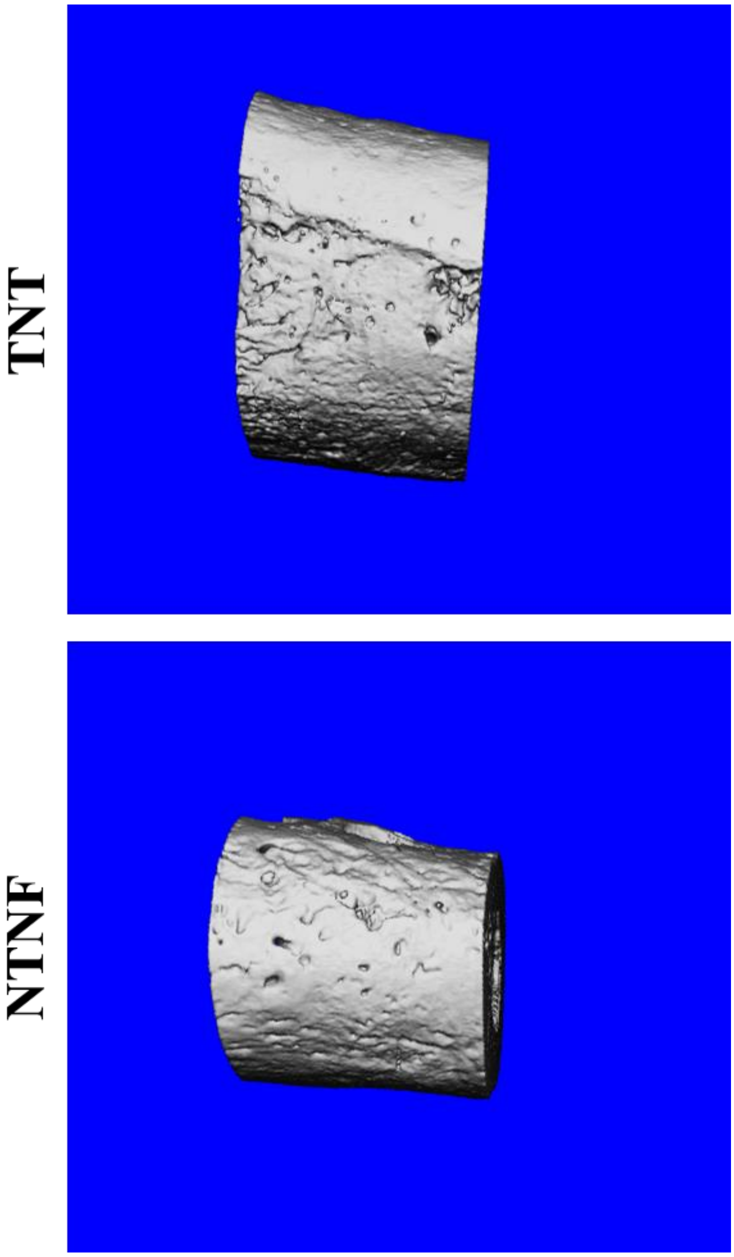

**Figure S8.** Micro-CT 3D reconstruction perspective view on the top and buccal of the implant at 12 weeks after implantation.

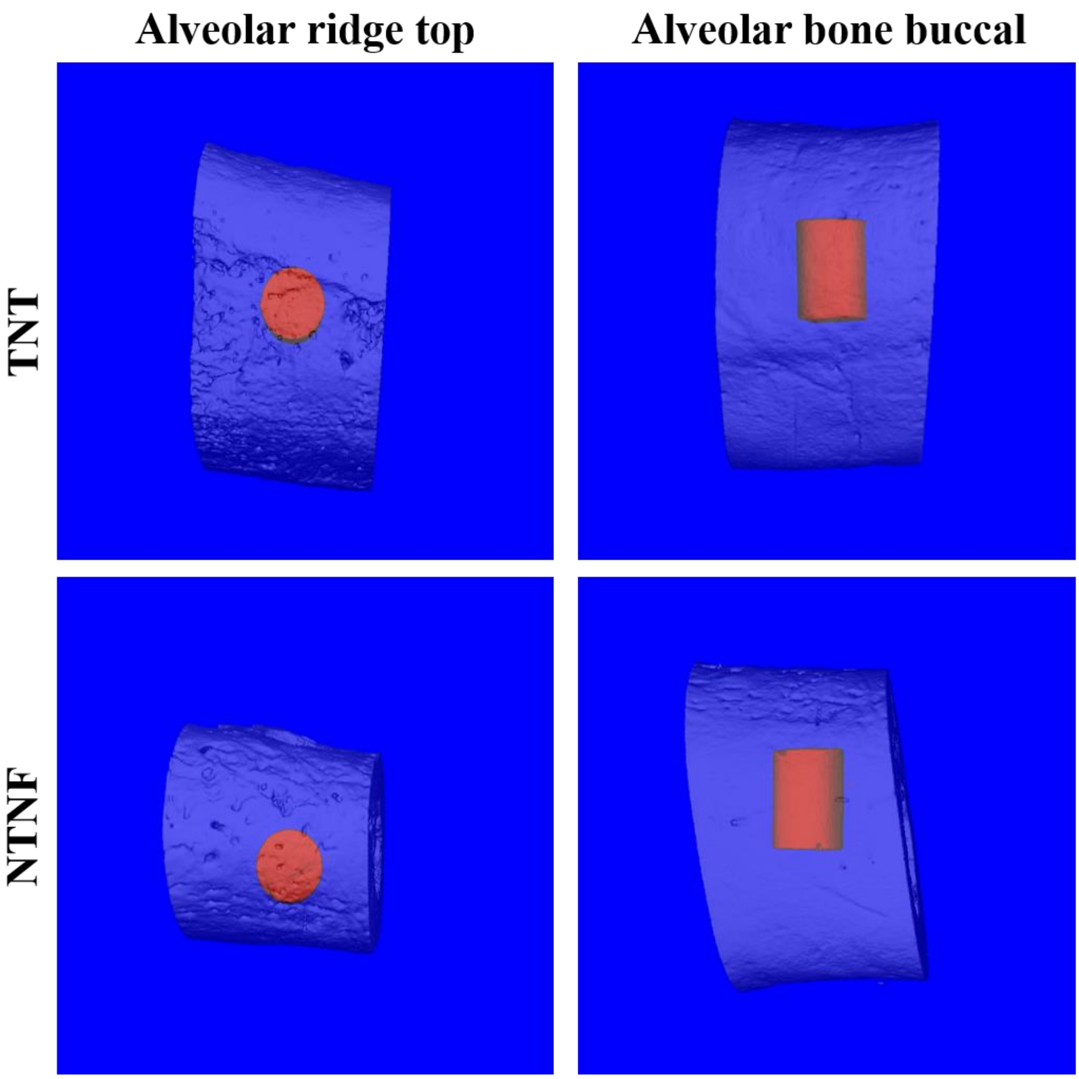

**Table S1.** The chemical compositions of the TNT and NTNF by EDS.

| % | TNT | NTNF |
|---|---|---|
| Ti | 56.8 | 84.9 |
| O | 35.7 | 11.8 |
| F | 6.6 | 0 |
| C | 0.9 | 3.3 |
